# Increased levels of synaptic proteins involved in synaptic plasticity after chronic intraocular pressure elevation and modulation by brain-derived neurotrophic factor in a glaucoma animal model

**DOI:** 10.1242/dmm.037184

**Published:** 2019-06-18

**Authors:** Hae-Young Lopilly Park, Si Won Kim, Jie Hyun Kim, Chan Kee Park

**Affiliations:** Department of Ophthalmology, Seoul St Mary's Hospital, College of Medicine, The Catholic University of Korea, Seoul 06591, Korea

**Keywords:** Retinal ganglion cell, Neurodegeneration, Synapse, IOP, BDNF

## Abstract

The dendrites of retinal ganglion cells (RGCs) synapse with the axon terminals of bipolar cells in the inner plexiform layer (IPL). Changes in the RGC dendrites and synapses between the bipolar cells in the inner retinal layer may critically alter the function of RGCs in glaucoma. The present study attempted to discover changes in the synapse using brain-derived neurotrophic factor (BDNF) after glaucoma induction by chronic intraocular pressure elevation in a rat model. Immunohistochemical staining revealed that the BDNF-injected group had a significant increase in the level of synaptophysin, which is a presynaptic vesicle protein, in the innermost IPL compared with the phosphate-buffered saline (PBS)-injected group. SMI-32, which is a marker of RGCs, was colocalized with synaptophysin in RGC dendrites, and this colocalization significantly increased in the BDNF-injected group. After the induction of glaucoma, the BDNF-injected group exhibited increases in the total number of ribbon synapses, as seen using electron microscopy. Expression of calcium/calmodulin-dependent protein kinase II (CaMKII), cAMP-response element binding protein (CREB) and F-actin, which are key molecules involved in synaptic changes were upregulated after BDNF injection. These initial findings show the capability of BDNF to induce beneficial synaptic changes in glaucoma.

## Abstract (precis)

**Summary:** Application of BDNF increased the expression of synaptic vesicle proteins in the inner retina via the *p*-Akt, CaMKII and CREB pathways, increasing F-actin in RGC dendrites.

## INTRODUCTION

Glaucoma is a neurodegenerative disease for which the pathological hallmark is retinal ganglion cell (RGC) death (Quigley et al., 1981). A number of studies have reported that a chronic elevation in intraocular pressure (IOP), which is a clinical hallmark of glaucoma, induces axonal degeneration and the apoptosis of RGCs (Quigley, 1999; Quigley et al., 1995; Garcia-Valenzuela et al., 1995). However, there are synaptic degenerations and changes in the size and morphology of the soma and dendrites of RGCs before the initiation of axonal degeneration and RGC apoptosis (Shou et al., 2003; Weber et al., 1998). Dendrites of RGCs are thought to respond and show compensative changes after injury, such as increasing dendritic receptive fields and developing new branches (Schwab, 1996; Dancause et al., 2005; Papadopoulos et al., 2002). A previous study from our research group found that RGCs exhibit a dendritic response as well as synaptic changes following chronic IOP elevation (Park et al., 2014). In that study, we found an increase in synaptophysin, which is a presynaptic vesicle protein, and a change in the morphological characteristics of the synapses. These findings suggest that there are biological attempts to modulate synaptic plasticity after elevations in IOP, and therefore, the next step will be finding a method to enhance synaptic plasticity following chronic IOP elevation in glaucoma.

Brain-derived neurotrophic factor (BDNF) has been extensively investigated and is known to contribute to synaptic development and the formation of connections between neurons during development (Zhong et al., 2015; Leal et al., 2014; Lu, 2003). Although there are studies showing synaptic rearrangements and dendritic shrinkage after glaucoma induction, BDNF application exhibited a delay in dendritic retraction (Agostinone and Di Polo, 2015; Binley et al., 2016). There was a study showing the role of BDNF after exercise through an AMP-activated protein kinase pathway, which prevented synapse elimination, but there are few studies focusing on the synaptic elements after BDNF application in the retina (Chrysostomou et al., 2016). Calcium/calmodulin-dependent protein kinase II (CaMKII) and cAMP-response element binding protein (CREB) are reported to have a role in synaptic plasticity (Kotaleski and Blackwell, 2010; Maren and Baudry, 1995; Evans, 2007). Previous investigations showed CaMKII as a mediator of synaptic plasticity in hippocampal and other neurons, through activation of the CREB pathway (Bustos et al., 2017). Several attempts, including BDNF application, were made to modulate the CaMKII and CREB pathway to promote synaptic plasticity and neurite outgrowth (Yan et al., 2016; Clarkson et al., 2015). However, there are only a few studies that have investigated the CaMKII and CREB pathway in relation to synaptic changes in RGCs and its modulation by BDNF in glaucoma. Thus, the present study investigated the effects of BDNF on the modulation of RGC synapses and the CaMKII-CREB pathway after chronic elevation in IOP.

## RESULTS

### BDNF application reduces retinal stress and RGC apoptosis by inducing BDNF expression and phosphorylation of Akt in the retina after IOP elevation

**Fig. 1.**
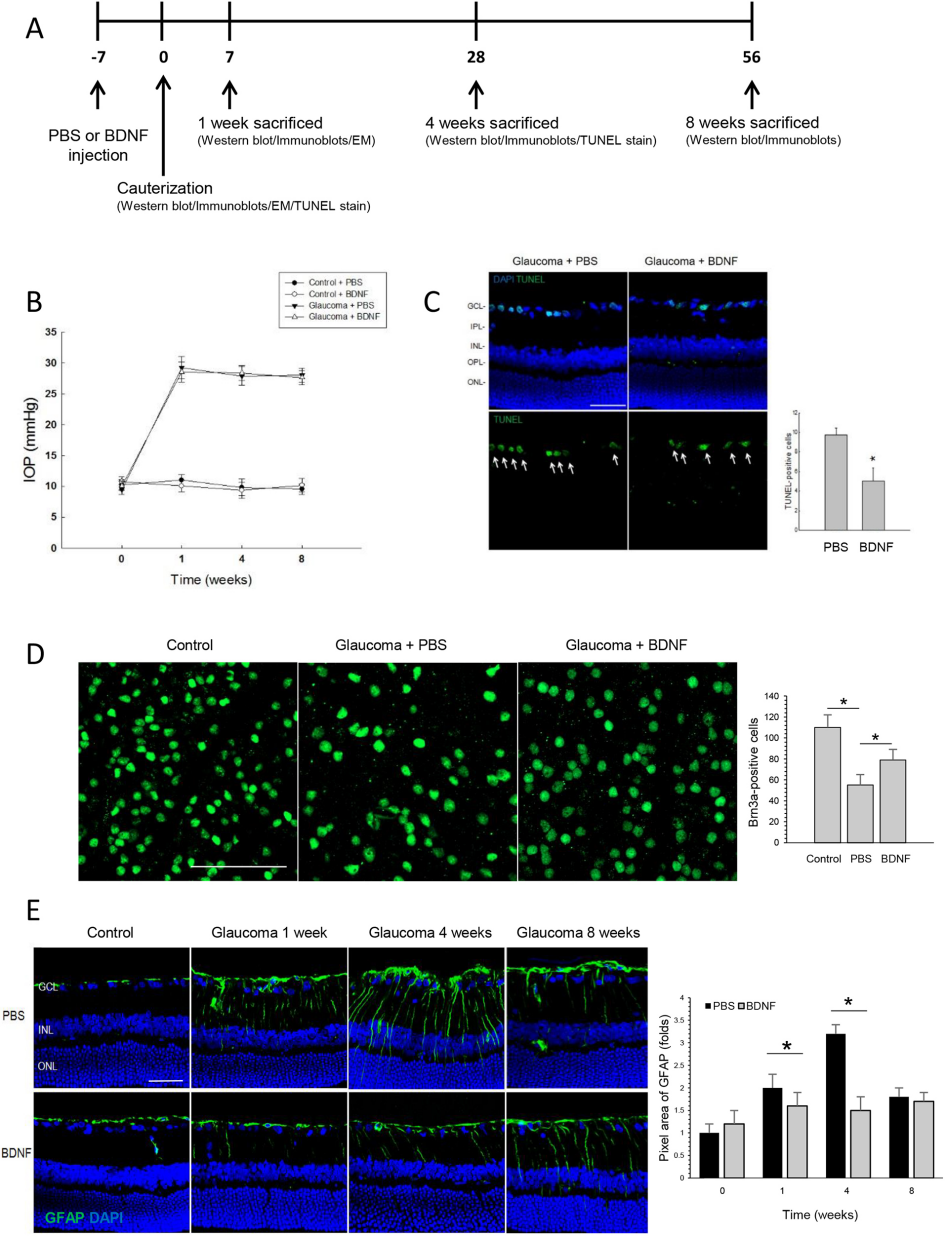
**Study scheme and results of BDNF application to RGC apoptosis and GFAP expression.** (A) Schematic of the experimental time points. (B) Verifying elevations in intraocular pressure (IOP) in a chronic hypertension model of glaucoma. Changes in IOP after the cauterization procedure are shown. The IOPs of the control (sham-operated) and cauterized eyes were measured at 0, 1, 4 and 8 weeks after cauterization. The IOP of the cauterized eyes remained elevated throughout the 8-week experimental period. The intravitreal injections of phosphate-buffered saline (PBS) or brain-derived neurotrophic factor (BDNF) did not influence IOP in either the control (sham-operated) or cauterized groups. For the IOP measurements, the PBS-injected group and the BDNF-injected group each included 3 animals at each time point; total *n*=24. (C) Confocal micrographs of the terminal deoxynucleotidyl transferase-mediated dUTP nick-end labeling (TUNEL) assay showing apoptotic retinal ganglion cells (RGCs) in the ganglion cell layer (GCL) at week 4 after cauterization. There was a significant decrease in TUNEL-positive RGCs in the BDNF-injected group compared with the PBS-injected group. For the TUNEL assay, the PBS-injected group and the BDNF-injected group each included 6 retinas at baseline and week 4; 10 sections per retina were analyzed; total *n*=48. Scale bar: 50 μm. *P<0.05. (D) Confocal micrographs of flat-mounted retinas stained for Brn3a, a specific RGC marker, at week 4 after cauterization. Brn3a-positive cells significantly decreased at 4 weeks after cauterization compared to baseline, which was significantly preserved after BDNF injection. For the flat-mount retina stain, the baseline control, the PBS-injected group and the BDNF-injected group each included 4 retinas; 24 sections per retina were analyzed; total *n*=12. Scale bar: 50 μm. *P<0.05. (E) Confocal micrographs of cross-sectional retinas stained for glial fibrillary acidic protein (GFAP). The immunoreactivity for GFAP increased throughout the inner retinal layers after the induction of glaucoma until week 4, but significantly decreased in the BDNF-injected group compared with the PBS-injected group. For the GFAP staining, the PBS-injected group and the BDNF-injected group each included 6 retinas at each time point; 10 sections per retina were analyzed; total *n*=48. Scale bar: 50 μm. *P<0.05.

One week prior to episcleral vein cauterization procedure, the experiment eyes of each rat received an intravitreal injection of BDNF (5 μg/10 µl) while the opposite eye received an intravitreal injection of phosphate-buffered saline (PBS) as control (Fig. 1A). Cauterization of the episcleral vein induced a sustained elevation of IOP throughout the entire 8-week experiment in the eyes that were analyzed (Fig. 1B). More specifically, 1 week after the surgery, there was a gradual increase in IOP from a basal value of 10.2±1.96 mmHg to 29.5±2.12 mmHg. The average IOP in the cauterized eye over the 8-week experimental period was 27.3±2.19 mmHg; control eyes that underwent sham surgery maintained normal IOP throughout the experiment. The intravitreal injections of BDNF and PBS did not result in differences in IOP in either the sham or glaucoma surgery groups.

After confirming IOP elevation, retinal stress and RGC death were evaluated by a terminal deoxynucleotidyl transferase-mediated dUTP nick-end labeling (TUNEL) assay and immunostaining of glial fibrillary acidic protein (GFAP), respectively. TUNEL staining revealed increased RGC apoptosis at 4 weeks after cauterization; however, the number of TUNEL-positive cells in the ganglion cell layer (GCL) significantly decreased after BDNF injection compared with the PBS-injected group (Fig. 1C). Additionally, Brn3a staining, a specific RGC marker, on flat-mounted retinas shows decreased Brn3a-positive cells in the GCL after 4 weeks of cauterization compared to the control. However, the number of Brn3a-positive cells in the GCL was significantly increased after BNDF application (Fig. 1D). Immunoreactivity for GFAP was elevated in the GCL, inner plexiform layer (IPL), inner nuclear layer and outer plexiform layer after 1 and 4 weeks following cauterization (Fig. 1E); however, increased GFAP expression was reduced throughout the inner retinal layers by intravitreal injections of BDNF after 1 and 4 weeks following cauterization.

**Fig. 2.**
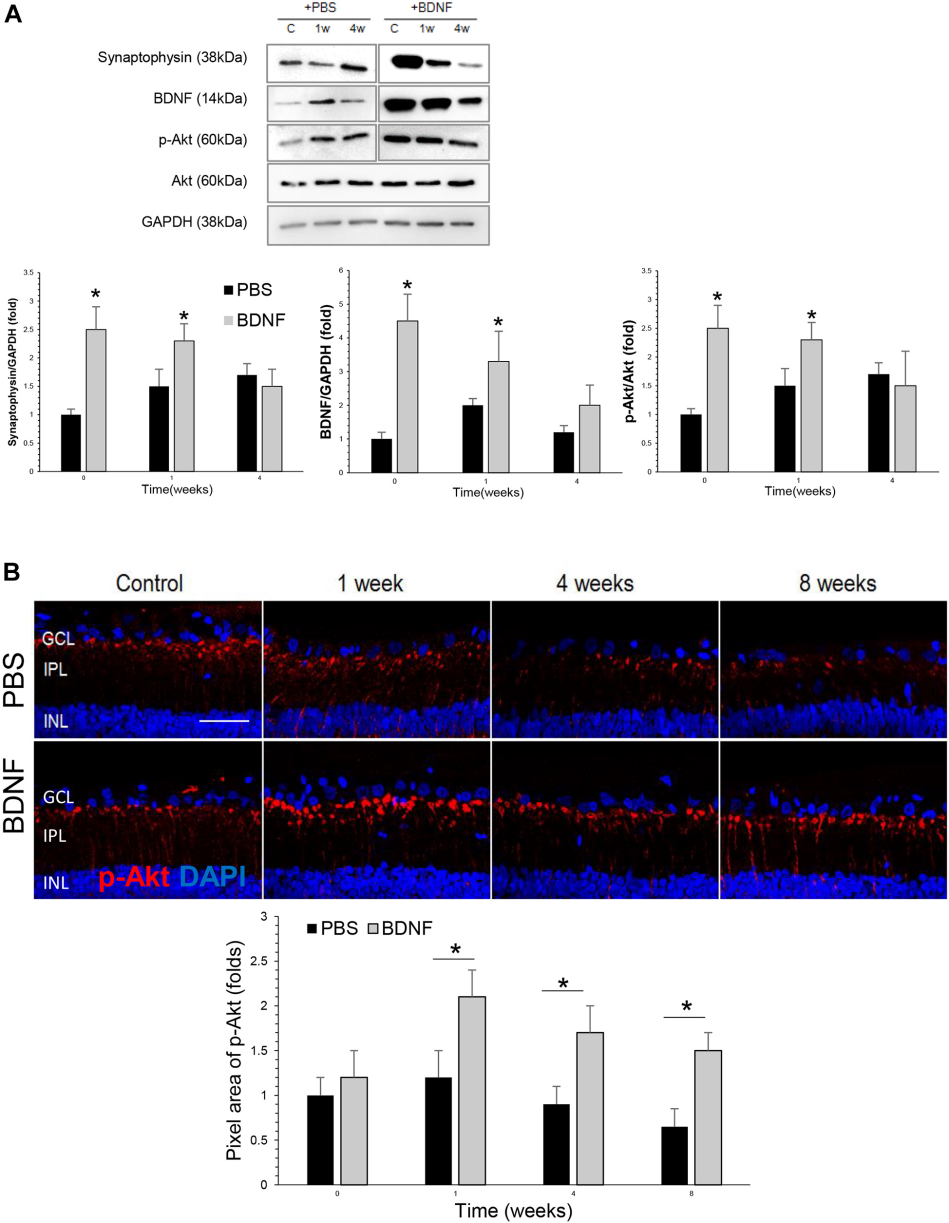
**Effects of BDNF application to expression of synaptic proteins and phosphorylated Akt.** (A) Results of western blot analysis of BDNF, synaptophysin and phosphorylated Akt. There were significant elevations in BDNF in the BDNF-injected group compared with the PBS-injected group at baseline and 1 week after cauterization. Additionally, there was a significant increase in the level of synaptophysin, which is a synaptic vesicle protein, and phosphorylated Akt in the retinas of the BDNF-injected group compared with the PBS-injected group at baseline and 1 week after BDNF-injection. For the western blot analysis, the PBS-injected group and the BNDF-injected group each included 6 retinas; total *n*=36. *P<0.05. (B) Confocal micrographs of retinal sections stained for phosphorylated Akt. In the BDNF-injected group, there was an increase in phosphorylated Akt levels in the innermost IPL compared with the PBS-injected group; this peaked at week 1 and lasted until week 8. For the *p*-Akt staining and quantification, the PBS-injected group and the BDNF-injected group each included 6 retinas at each time point; 10 sections per retina were analyzed; total *n*=48. Scale bar: 50 μm. *P<0.05.

Western blot analysis revealed that, compared with the PBS-injected group, the BDNF-injected group had significantly higher levels of BDNF in the retina at baseline and 1 week after cauterization (Fig. 2A), which was 1 week and 2 weeks after the BDNF injection, respectively. Synaptophysin and phosphorylated Akt (*p*-Akt) protein levels were significantly elevated at baseline (1 week after the injection) and 1 week (2 weeks after injection) in the BDNF-injected group compared to the PBS-injection group. Thereafter, synaptophysin and *p*-Akt protein level decreased to basal level at week 4 compared to the PBS-injected group in the western blot analysis.

The immunoreactivity of *p*-Akt increased in the innermost IPL and peaked at 1 week after cauterization (Fig. 2B). Comparison between the BDNF- and PBS-injected groups shows that BDNF injection significantly increased expression of *p*-Akt in the innermost IPL at 1, 4 and 8 weeks after cauterization.

### Increased number of presynaptic vesicles co-stained with RGC dendrites after the application of BDNF

**Fig. 3.**
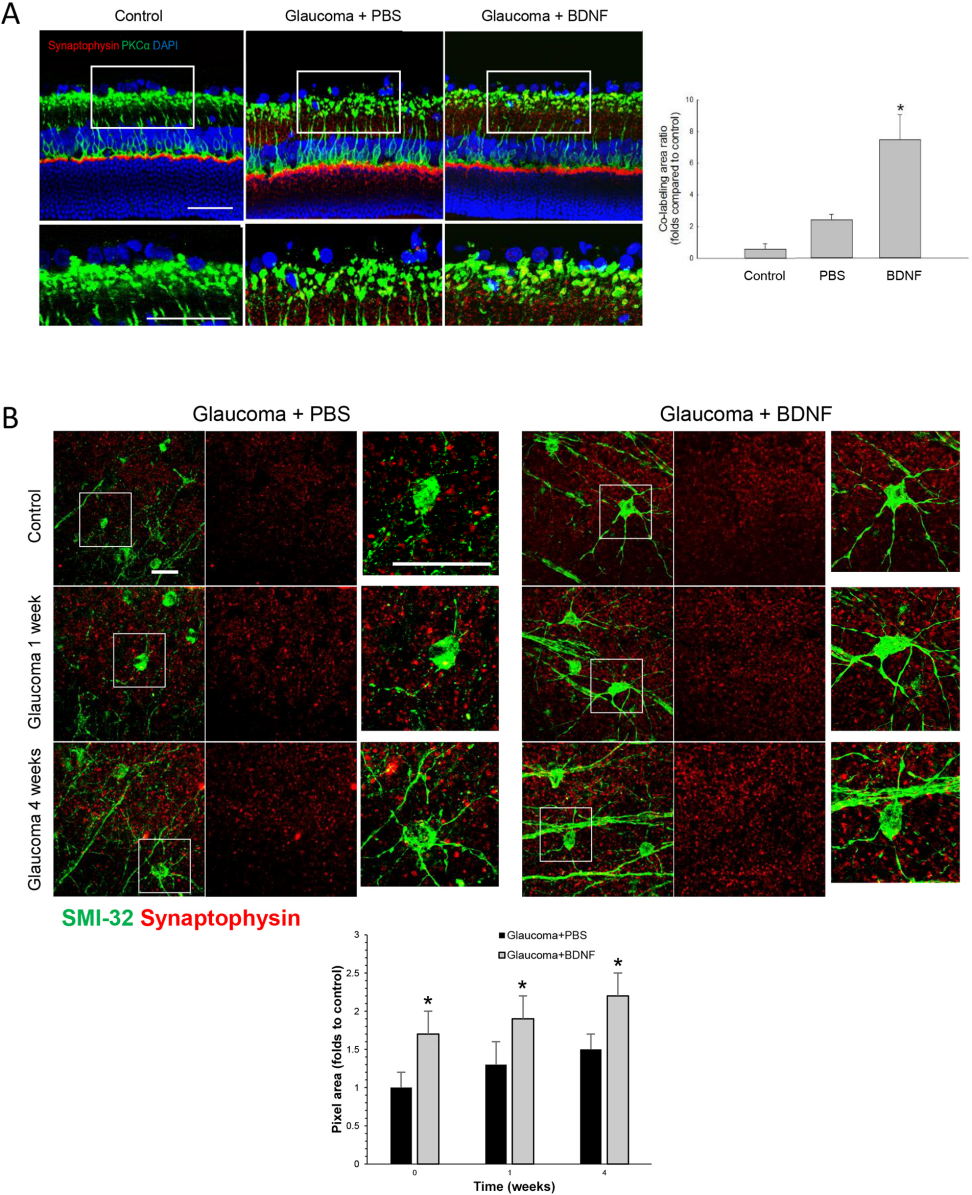
**Effects of BDNF application to expression of synaptophysin and dendritic structures.** (A) Confocal micrographs of retinal sections double-stained for synaptophysin, which is a marker of synaptic vesicles, and protein kinase C-alpha (PKCα), which is a marker of bipolar cells, 1 week after cauterization. In the PBS-injected group, synaptophysin was expressed in the innermost inner plexiform layer (IPL) and the outer plexiform layer (OPL) at week 4 after the induction of glaucoma; these levels increased after the injection of BDNF. Furthermore, the expression of synaptophysin and the co-staining between synaptophysin and PKCα both increased in the innermost IPL in the BDNF-injected group compared with the PBS-injected group. Magnified confocal micrographs of the innermost IPL showed that the co-stained area significantly increased in the BDNF-injected group compared with the PBS-injected group. For the synpatophysin staining and quantification, the PBS-injected group and the BDNF-injected group each included 6 retinas at baseline and week 1; 10 sections per retina were analyzed; total *n*=18. Scale bars: 50 μm. (B) Confocal micrographs of flat-mounted retinas double-stained for synaptophysin, which is a marker of synaptic vesicles, and SMI-32, which is an RGC marker, focused on the border of the ganglion cell layer (GCL) and the IPL. SMI-32 staining was observed in both the soma and dendrites of the RGCs. Synaptophysin immunoreactivity increased at weeks 1 and 4 after the induction of glaucoma compared with week 0 (control). After the application of BDNF, SMI-32-positive RGCs increased at weeks 0 and 1 along with increased synaptophysin immunoreactivity at weeks 1 and 4. Additionally, the SMI-32 immunoreactivity revealed increased and thickened RGC dendrites at weeks 0, 1 and 4 after the BDNF injection. For the flat-mount synpatophysin staining and quantification, the PBS-injected group and the BDNF-injected group each included 6 retinas at baseline, week 1 and week 4; 10 sections per retina were analyzed; total *n*=36. Scale bars: 50 μm.

The presynaptic vesicle proteins were assessed by immunostaining for synaptophysin. Immunoreactivity of synaptophysin and co-labeling with protein kinase C-alpha (PKCα), which is a marker of bipolar cells, revealed that increased expression of synaptophysin occurred within the bipolar cells in the innermost IPL (Fig. 3A). The location was similar to the *p*-Akt expression in the innermost IPL. After BDNF injection, the co-labeling of synaptophysin and PKCα significantly increased in the IPL compared to the PBS-injection group after 1 week of cauterization.

To investigate dendritic morphology and presynaptic vesicle protein expression, flat-mount preparations of the retinas were analyzed via an immunostaining procedure for markers of RGCs. From the flat-mounted retina, six *z*-stack images of 0.5-μm intervals were averaged, resulting in a scan of 2.5 μm thickness starting from the GCL surface. SMI-32, which is a marker of neurofilaments, stains both the soma and dendrites of the RGCs. In the PBS-injected group, the colocalization between SMI-32 and synaptophysin significantly increased at 1 and 4 weeks after cauterization compared to baseline in the GCL (Fig. 3B). Compared with the PBS-injected group, the colocalization between SMI-32 and synaptophysin further increased in the BDNF-injected group at 1 and 4 weeks after cauterization. The dendritic morphology of the RGCs in the BDNF-injected group shows an increased number of dendritic branches compared to the PBS-injected group at baseline and 1 week after cauterization (Fig. 3B, magnified images).

### The application of BDNF increased the number of ribbon synapses and expression of synaptic spines

**Fig. 4.**
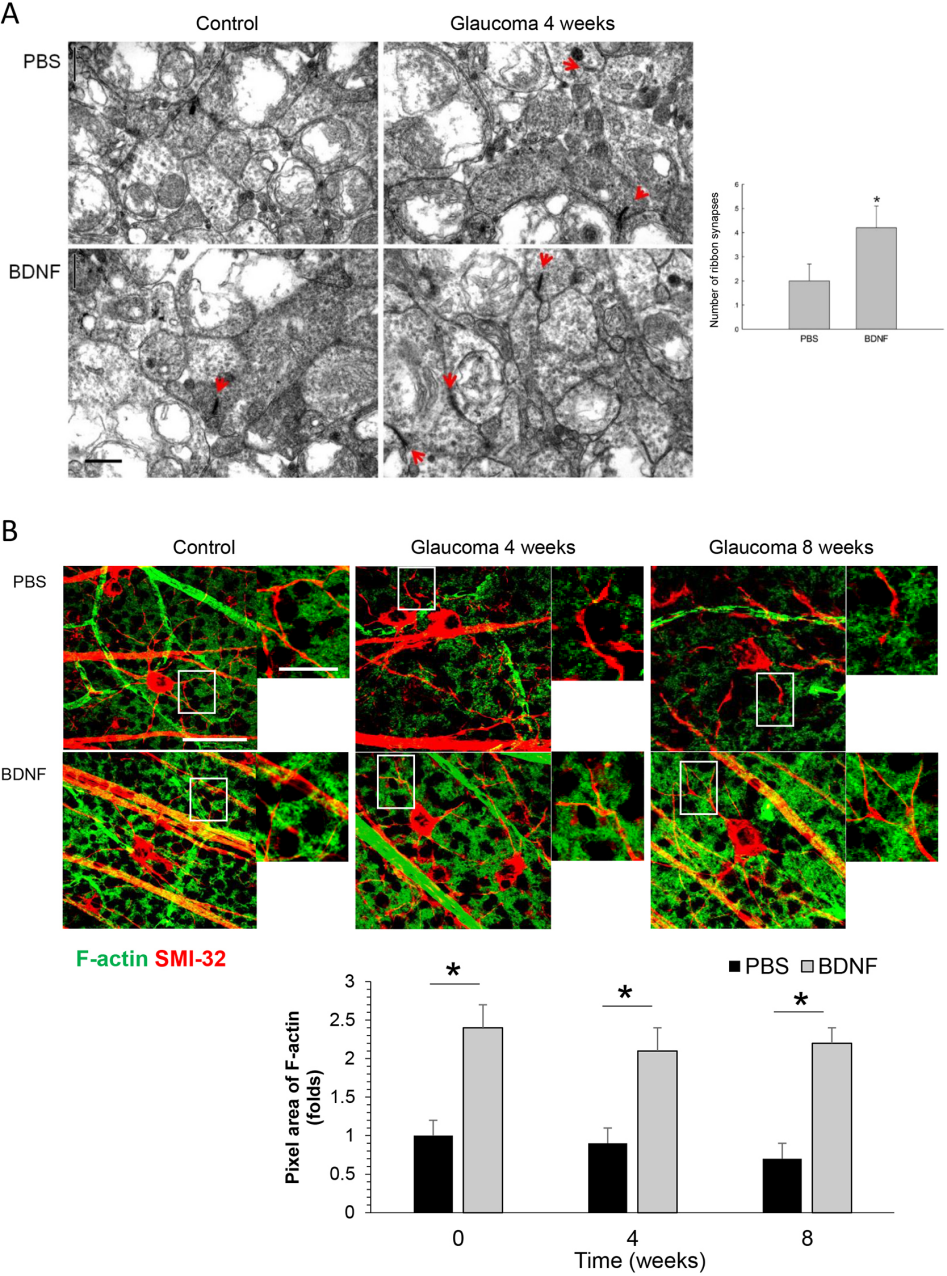
**Effects of BDNF application to ribbon synapses and F-actin expression.** (A) Transmission electron micrographs depicting ribbon synapses between RGCs and bipolar cells in the IPL. Four weeks after the induction of glaucoma, there were increases in the number of synapses (red arrow) in the BDNF-injected group compared with the PBS-injected group. For the electron microscopic analysis, the PBS-injected group and the BDNF-injected group each included 6 retinas at baseline and week 4; 10 sections per retina were analyzed; total *n*=24. Scale bar: 0.5 μm. *P<0.05. (B) Confocal micrographs of flat-mounted retinas double-stained for F-actin, the final product of the activated CaMKII and CREB pathway that composes the synaptic spine of dendrites, and SMI-32, which is an RGC marker, focused on the border of the GCL and the IPL. BDNF injection increases F-actin expression around the dendrites of RGCs compared to the PBS-injection group at 4 and 8 weeks after cauterization. For the flat-mount F-actin staining, the PBS-injected group and the BDNF-injected group each included 6 retinas at each time point; 10 sections per retina were analyzed; total *n*=36. Scale bars: 50 μm. *P<0.05.

The number of ribbon synapses in the IPL was quantified in 10 fields per 6 retinal sections from 6 eyes in each group using transmission electron microscopy (Fig. 4A). The numbers were 6.5/50 μm^2^ and 2.1/50 μm^2^ at baseline and 4 weeks after cauterization, respectively, in the PBS-injected group, and 5.2/50 μm^2^ and 4.5/50 μm^2^ at baseline and 4 weeks after cauterization, respectively, in the BDNF-injected group. The number of ribbon synapses were significantly increased in the BDNF-injected group at 4 weeks after cauterization compared to the PBS-injected group.

F-actin (filamentous actin) is the final product of activated CaMKII and CREB, and composes the synaptic spine of dendrites. Immunostaining with anti-F-actin and -SMI-32 in retinal whole mounts focused on the GCL shows that BDNF injection increases F-actin expression around the dendrites of RGCs compared to the PBS-injection group at 4 and 8 weeks after cauterization (Fig. 4B).

### Changes in the molecular pathway involved in synaptic plasticity after application of BDNF

**Fig. 5.**
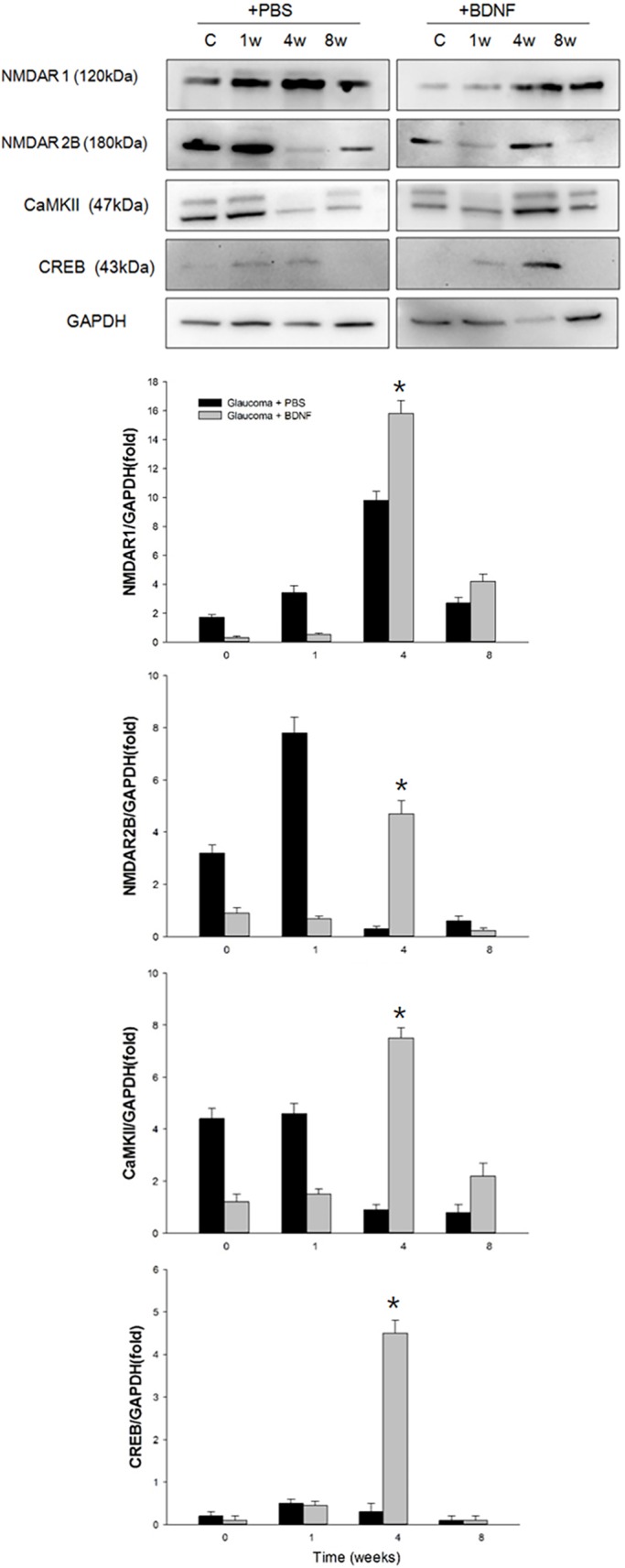
**Effects of BDNF application to proteins related to synaptic plasticity.** Results of western blot analysis of NMDA receptor 1 (NMDA1), NMDA2B, *p*-CaMKII and *p*-CREB. There were significant elevations in NMDA1, NMDA2B, *p*-CaMKII and *p*-CREB in the BDNF-injected group compared with the PBS-injected group at 4 weeks after cauterization. For the western blot analysis, the PBS-injected group and the BNDF-injected group each included 6 retinas at each time point; total *n*=48. The bar represents the mean±s.d. and a Student's *t*-test was used for statistical evaluation. *P<0.05.

**Fig. 6.**
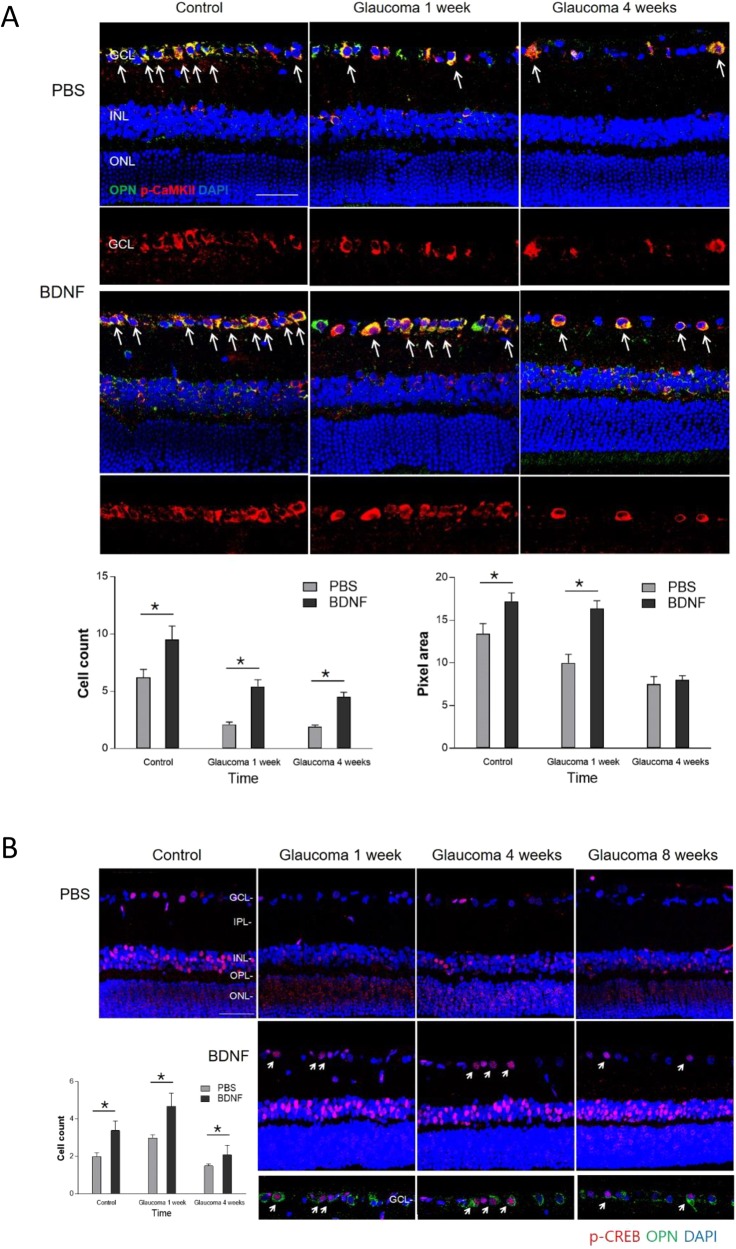
**Effects of BDNF application to expression of CaMKII and CREB.** (A) Confocal micrographs of retinal sections stained for *p*-CaMKII and optineurin (OPN), which is a ganglion cell marker. The double-stained cells in the GCL are marked with white arrows. BDNF-injected retinas showed a significant increase in double-stained cells in the GCL at all experimental time points. The pixel areas for *p*-CaMKII in the GCL showed significant upregulated expression at baseline and 1 week after cauterization. For the immunohistochemical staining, the PBS-injected group and the BDNF-injected group each included 6 rats at each time point; total *n*=36. Scale bar: 50 μm. *P<0.05. (B) Confocal micrographs of retinal sections stained for *p*-CREB and OPN. The double-stained cells in the GCL are marked with white arrows. After cauterization, expression of CREB decreased in the GCL at all experimental time points compared with the baseline. However, a significant increase in expression of CREB was found after BDNF injection and the double-stained cells with CREB and OPN in the GCL significantly increased at baseline, 1 week and 4 weeks after cauterization. For both p-CaMKII and p-CREB staining, the PBS-injected group and the BDNF-injected group each included 6 retinas at each time point; total *n*=48. Scale bar: 50 μm. *P<0.05.

To find out the molecular pathway involved in the changes in synapses after IOP elevation and BDNF application, western blot analysis for proteins of N-methyl-D-aspartate receptor (NMDAR)1 and 2B, phosphorylated CaMKII (*p*-CaMKII) and phosphorylated CREB (*p*-CREB) were performed (Fig. 5). After BDNF injection, a significant increase in NMDAR1 and 2B, *p*-CaMKII, and *p*-CREB were found in the glaucomatous retina at week 4 compared to the PBS-injected group. Immunostainings for *p*-CaMKII and *p*-CREB were significantly increased in the RGCs, which were stained with optineurin as a ganglion cell marker, after BDNF injection compared to the PBS-injected group throughout the experimental periods (Fig. 6A,6B). The number of co-labelling RGCs with *p*-CaMKII and optineurin (OPN) significantly increased at all experimental time points in the BDNF-injected group compared with the PBS-injected group. The pixel area of *p*-CaMKII in the GCL was significantly increased in the BDNF-injection group compared to the PBS-injected group at baseline and 1 week after cauterization (Fig. 6A). The number of cells stained with *p*-CREB in the GCL showed a significant increase after BDNF-injection at 1, 4 and 8 weeks after cauterization (Fig. 6B).

## DISCUSSION

The present study demonstrated that the levels of synaptic vesicle proteins in RGCs and bipolar cells increased after the application of BDNF in a rat model of glaucoma using chronic elevation in IOP. In the BDNF-injected group, the expression of synaptophysin increased in both the bipolar cells and RGC dendrites, and the number of total ribbon synapses increased in the IPL. These changes peaked at week 1, which was 2 weeks after the administration of a single BDNF injection, and decreased thereafter. The innermost IPL, where the synaptic vesicle proteins primarily increased, also exhibited an increase in the levels of phosphorylated Akt, which is downstream of BDNF receptor signaling, after the BDNF injection. The main components of the pathway involved in synaptic plasticity to form dendritic spines, which are the activated input structure for synaptic transmission, CaMKII and CREB, were elevated in the RGCs after BDNF injection. Expression of the final product of the CaMKII-CREB pathway, F-actin, was increased around the dendrites of RGCs after BDNF injection. These findings indicate that the application of BDNF can increase attempts to form synapses between RGCs and bipolar cells, and enhance synaptic plasticity after chronic IOP elevation.

Changes in the dendritic structure and morphology of RGCs occur prior to cell death in the glaucomatous eye; therefore, the present study aimed to investigate dendritic changes and synapses in greater detail. Early changes in RGC dendritic structure may have critical consequences for synaptic efficacy and may underlie functional deficits prior to RGC loss in patients with glaucoma (Weber and Harman, 2005). Our research group previously identified changes in synaptic vesicle proteins and the characteristics of the ribbon synapse after the induction of glaucoma (Park et al., 2014). In the present study, analyses of synaptophysin revealed that presynaptic proteins in the bipolar cells in the IPL gradually increased throughout the 8-week experimental period. Although the total number of synapses decreased due to significant RGC loss, there appeared to be attempts to increase the number of synaptic proteins and structures in the inner layers of the retina after chronic IOP elevation. Using electron microscopy, the present study identified immature and newly formed ribbon synapses in the inner retinal layers, which suggests that there is a compensatory mechanism for the restoration of synaptic connections between RGCs and bipolar cells following RGC apoptosis. The present findings indicate that this process can be modulated by the application of BDNF.

The present study also found that, in addition to synaptic changes, BDNF also modulated alterations in dendritic structure. In animal models of glaucoma using elevated IOP, the dendritic structure of the RGCs are either maintained or gain greater dendritic complexity (in a few select RGCs) compared with the control eyes (Kalesnykas et al., 2012). In the present study, the application of BDNF resulted in an increased number and thickening of the dendritic branches at each time point relative to the PBS-injected group. These results suggest that BDNF can modulate the morphological plasticity of RGC dendrites (Morgan et al., 2006). F-actin, which is the component of dendritic spines, increased around the RGC dendrites, indicating synaptic enhancement or synaptic plasticity.

A number of studies have shown that BDNF is vital for maintaining the health of RGCs in the retina and for protecting RGCs from insult-induced apoptosis (Unoki and LaVail, 1994; Peinado-Ramon et al., 1996). Among the wide variety of neurotrophic factors, BDNF is considered to be the most potent survival factor for injured RGCs (Jelsma et al., 1993; Mansour-Robaey et al., 1994; Perez and Caminos, 1995) because it promotes neuronal survival in cell cultures (Thanos and Vanselow, 1989; Johnson et al., 1986), stimulates the growth of neurites from regenerating RGCs (Cohen-Cory and Fraser, 1995), and significantly reduces axotomy-induced damage of the optic nerve and RGCs *in vivo* (Carmignoto et al., 1989; Mey and Thanos, 1993). Additionally, BDNF plays a role in the regulation of pre- and post-synaptic proteins, the enhancement of synaptic transmission, and increases in synaptic vesicle docking in the developing brain (Pascual et al., 2001; Patterson et al., 1996; Kang and Schuman, 1995). Furthermore, the BDNF protein and its downstream tyrosine receptor kinase B receptor signaling factors, including Akt, have been consistently reported to contribute to synapse formation during development (Jia et al., 2008). Endogenous BDNF could be stimulated after glaucoma induction. As shown in our western blot analysis, there is an increase in BDNF expression at week 1 after glaucoma induction in the PBS-injection group. The synaptic plasticity after application of BDNF could be a result of both endogenous and exogenous BDNF.

In the present study, there was an increase in the level of synaptic vesicle proteins and docked synaptic vesicles after the BDNF injection. Furthermore, the innermost IPL exhibited an increase in phosphorylated Akt levels after the early phase of the BDNF injection, which may be explained by the role of BDNF in synapse enhancement (Kim and Park, 2005; Kim et al., 2007). Expression of CaMKII and CREB increased in the RGCs after BDNF injection until 4 weeks after IOP elevation. This represents an additional potential therapeutic application for BDNF via its enhancement of synaptic plasticity in the adult retina. In particular, NMDAR1 and 2B were increased after 4 weeks of IOP elevation in the BDNF-injected group. NMDAR contributes to synaptic plasticity that involves CaMKII and CREB phosphorylation and is critical for synaptic transmission (Riccio and Ginty, 2002; Lau and Zukin, 2007). From our study, BDNF increases pathways involving NMDAR and its downstream pathways to maintain synapses and increase synaptic plasticity of the RGCs.

However, the effects of a single BDNF injection were transient and lasted for only approximately 2 weeks. The exogenous application of BDNF promoted RGC survival in a rat model of chronic hypertension (Ko et al., 2001), and intravitreal injections of BDNF reportedly slow RGC loss and support the survival of RGCs for up to 1 week after axotomy (Mansour-Robaey et al., 1994; Clarke et al., 1998). However, these previous studies also demonstrated the short half-life and temporal effects of BDNF. In the present study, the BDNF expression was shown to be increased at 1-2 weeks after BDNF injection compared to the PBS-injected group. However, BDNF was not detected at week 4, which was 5 weeks after BDNF injection. Synaptophysin expression also followed this pattern. In our previous study (Park et al., 2014), synaptophysin expression gradually increased with glaucoma induction until 8 weeks after glaucoma induction. This pattern was also shown in the present study and BDNF injection further increased synaptophysin expression at baseline and 1 week after glaucoma induction, which did not last at week 4. Repeated injections of BDNF or another type of delivery system may prolong the effects of this neurotrophic factor and should be further investigated to determine its application as a neuroenhancement therapy in glaucoma due to its ability to strengthen the synapse in the inner retina.

### Conclusions

The present study demonstrated that the application of BDNF increased the expression of synaptic vesicle proteins in the inner retina after the induction of glaucoma. Additionally, BDNF increased the total number of synapses between the RGCs and bipolar cells in the glaucomatous retina. Involved pathways were *p*-Akt, CaMKII and CREB, which increased F-actin in the RGC dendrites. These initial findings regarding the capability of BDNF to induce beneficial synaptic changes may aid in the development of neuroenhancement techniques that can be used to treat synaptic dysfunction in glaucoma.

## MATERIALS AND METHODS

### Animals

The present study included adult male Sprague-Dawley rats that were 7- to 8-weeks old and weighed 250-300 g. A total of 130 rats underwent surgery. A total of 16 rats failed to show IOP elevation throughout the experimental period and the remaining 228 eyes from 114 rats were used. A total of 96 eyes from 48 rats were used for immunohistochemical and TUNEL stains. A total of 12 eyes from 6 rats were used for flat-mount retina preparation and immunohistochemical stains. A total of 24 eyes from 12 rats were used for electron microscopy analysis. A total of 96 eyes from 48 rats were used for western blot analysis. All experimental procedures complied with The Association for Research in Vision and Ophthalmology (ARVO) statement for the Use of Animals in Ophthalmic and Vision Research and the National Institutes of Health (NIH) Guide for the Care and Use of Laboratory Animals (NIH Publications, no. 80-23, revised 1996). The IRB of the Catholic Ethics Committee of the Catholic University of Korea, Seoul approved the methods of this study. All efforts were made to minimize the number of animals used and any suffering they may have experienced.

Prior to the cauterization surgery, the rats were anesthetized with intraperitoneal injections of tiletamine-zolazepam (50 mg/kg body weight; Zoletil-Virbac) and xylazine hydrochloride (15 mg/kg; Rompun; Bayer, Leuverkeusen, Germany). Subsequently, three episcleral veins were cauterized using a standard technique as previously described (Kim and Park, 2005). Briefly, prior to the procedure, IOP was measured with a rebound tonometer (TonoLab, Icare, Finland) and then a small conjunctival incision was made in each quadrant at the limbus and the extraocular muscles were isolated. Four major limbal draining veins were identified based on their deep location under the rectus muscles, relative immobility, large caliber and branching pattern. Of these, 3 episcleral veins, specifically 2 dorsal episcleral veins from under the superior rectus muscle and 1 temporal episcleral vein from under the lateral rectus muscle, were cauterized using a surgical microscope (Olympus, Tokyo, Japan) and a cautery with a 30-gauge tip (Bovie Co., Melville, NY, USA) to avoid possible damage to the neighboring sclera. Planar ophthalmoscopy was used to confirm the normal perfusion of the retina.

After the surgery, chloramphenicol eye drops and oxytetracycline ointment were applied to the eyes. Only the eyes that did not suffer scleral burns with subsequent necrosis or any surgical complications were used for the present study. IOP was measured directly and carefully with a tonometer (Tono-Pen) after topical anesthetization with a proparacaine hydrochloride ophthalmic solution (Alcane; Alcon Laboratories, Fort Worth, TX, USA); the animals were kept as calm as possible to minimize any effects on the IOP readings. The experimental analyses were performed at 1, 4 and 8 weeks after the cauterization, and any eyes that did not show sustained IOP throughout the 8-week experimental period were excluded from the analyses.

### Intravitreal injections

Prior to the surgery, the rats were anesthetized with intraperitoneal injections of tiletamine-zolazepam (50 mg/kg; Zoletil-Virbac) and xylazine hydrochloride (15 mg/kg; Rompun, Bayer). Next, the pupils were dilated with eye drops containing 0.5% tropicamide and 2.5% phenylephrine hydrochloride, and the ocular surface was anesthetized with a topical application of 0.5% proparacaine hydrochloride (Alcane). One week prior to the episcleral vein cauterization, a microinjection needle was used to deliver either 5 μg/10 μl of BDNF or 10 μl of PBS intravitreally.

### Tissue preparation

For the immunohistochemical analyses at each time point, the eyes were quickly enucleated and dissected. The posterior eye cups were placed in chilled fixative [4% paraformaldehyde in 0.1 M phosphate buffer (PB), pH 7.4]. Then, the isolated retinas were immersed in the same fixative for 2 h at 4°C. After several washes, the fixed retinas were cryoprotected in 30% sucrose containing 0.1 M PB for 6 h at 4°C and then stored in the same buffer at −70°C. For the electron microscopy analyses, the retinal tissues were fixed in glutaraldehyde. For the western blot analysis, the retinal tissues were quickly dissected, frozen in liquid nitrogen and stored at −70°C.

### TUNEL assay

The presence of apoptotic cells was evaluated using TUNEL staining. For this procedure, the retinas were dissected from the choroid and the central portion of the superior nasal quadrant (1.5 mm from the optic disc) was trimmed into small pieces. Next, cryosections of the retina (50 μm) were immersed in 4% paraformaldehyde and washed with PBS. The tissue was stained using the TUNEL method according to the manufacturer's protocol (In Situ Cell Death Detection Kit; Roche Applied Science, Indianapolis, IN, USA). The next day, the sections were washed several times in PBS, incubated with goat anti-rabbit Alexa^®^ Fluor 546 (Molecular Probes, Eugene, OR, USA), washed again several times in PBS, immersed in 0.1 M PB for 30 min, and then mounted using VECTASHIELD^®^ Mounting Medium with 4′,6-diamidino-2-phenylindole (DAPI; H-1200; Vector Laboratories, Burlingame, CA, USA). The sections were washed, coverslipped and examined using confocal laser scanning microscopy (Zeiss, Germany).

For the quantification of TUNEL-positive cells, positive immunohistochemically stained cells in the GCL were counted in each retinal section (40× magnified cross-sectional images of the retina). TUNEL assay was performed in controls and at week 4 in both sham-operated controls and cauterized eyes (total of *n*=24). In each group, 6 retinas from 6 eyes were analyzed with 10 retinal sections per retina. Mean value of the count from 10 retinal sections was used in the statistical analysis.

### Transmission electron microscopy

The electron microscopy analyses were conducted using retinal sections from vein-cauterized and sham-operated subjects 4 weeks after the surgery (6 retinas per group); 10 fields of each eye were examined. Retinal sections (100 μm) were cut with a vibratome, post-fixed with 4% glutaraldehyde in 0.1 mmol/l cacodylate buffer (pH 7.4) for 1 h, and then fixed with 1% osmium tetroxide in 0.1 mmol/l cacodylate buffer for 2 h. After a rinse with distilled water, the sections were treated overnight with 1% aqueous uranyl acetate, dehydrated in increasing concentrations of ethanol solutions up to 100%, further dehydrated with dry acetone, and then embedded using Durcupan™ ACM. Ultrathin sections (0.1 μm) were cut and mounted on Formvar-coated slot grids, stained with 3% lead citrate and examined with a Zeiss transmission electron microscope (Zeiss). The ribbon synapses in the IPL between RGCs and bipolar cells were counted in 50 micrographs (2500 μm^2^ total) for every 10 fields in each retina. For the quantification, the average of counts from 10 fields in 6 retinas were used in the statistical analysis.

### Immunohistochemistry

The retinal expression of synaptophysin was assessed to evaluate the presence of synaptic vesicles. For the fluorescence staining, the samples were pre-embedded in 3% agar in deionized water and then sliced with a vibratome (50 μm) and washed several times in PBS. The sections were incubated in 10% normal donkey serum in PBS for 1 h at room temperature to block nonspecific binding activity and then incubated in anti-rabbit synaptophysin (Cell Signaling Technology, Danvers, MA, USA) overnight at 4°C. After several washes with PBS, the sections were incubated with goat anti-rabbit Alexa^®^ Fluor 546 (Molecular Probes).

For the double-labeling studies, the sections were incubated with either anti-mouse PKCα (Santa Cruz Biotechnology, Santa Cruz, CA, USA), SMI-32 (Covance, Emeryville, CA, USA), OPN (Santa Cruz Biotechnology), *p*-Akt (Cell Signaling Technology, Boston, MA, USA), *p*-CaMKII (Santa Cruz Biotechnology), *p*-CREB (Cell Signaling Technology), F-actin (Cell Signaling Technology), NMDAR1 (Cell Signaling Technology) and NMDAR2B (Cell Signaling Technology) in 0.1 M PBS containing 0.5% Triton X-100 overnight at 4°C. The sections were then rinsed with 0.1 M PBS for 30 min and incubated with goat anti-mouse Alexa^®^ Fluor 488 (Molecular Probes) for 90 min at room temperature. After further washing in 0.1 M PB for 30 min, the sections were mounted using VECTASHIELD^®^ Mounting Medium with DAPI (H-1200; Vector Laboratories). The slides were washed, covered with coverslips and examined by confocal laser scanning microscopy (Zeiss LSM 510, Carl Zeiss Co. Ltd). For flat-mounted retinas, images were taken starting from the surface of the GCL. Averages of 6 *z*-stack images of 0.5-μm intervals were taken, resulting in a 2.5-μm thickness scan starting from the GCL surface.

For the quantification of immunohistochemical images, images of the target protein were converted into a binary slab through the mean threshold algorithm of ImageJ software (http://rsb.info.nih.gov/ij/index.html), which automatically computes the threshold value as the mean of the local grayscale distribution. Each binarized 8 bit image was converted into a red, green, blue color model and then spilt into 3 channels (red, green and blue). After assigning white pixels as target protein and black pixels as background, the expression level of target protein in the GCL was calculated using ImageJ. For the quantification, 6 retinas from 6 eyes were analyzed with 10 retinal sections per each group. The mean value of the count from 10 retinal sections was used in the statistical analysis.

### Brn3a staining and quantification from flat-mount retina

Immediately after sacrifice, the superior side of each eye was marked for orientation, and both eyes were enucleated. The anterior segments were removed and the posterior segments were fixed in 4% paraformaldehyde in 0.1 M PB, pH 7.4, for 30 min. The retina was then isolated, divided into 4 equal quadrants and flat-mounted on slides. The retinas were incubated in 0.5% Triton X-100 and then with 10% normal donkey serum in PBS overnight at 4°C. After several PBS washes, tissues were incubated with anti-mouse Brn3a (Santa Cruz Biotechnology) overnight at 4°C. After several washes with PBS, the sections were incubated with goat anti-rabbit Alexa^®^ Fluor 546 (Molecular Probes). Brn3a-positive RGCs were counted as previously reported (Levkovitch-Verbin et al. 2003). Briefly, each retinal quadrant was divided into central, middle and peripheral regions (1, 2 and 3 mm from the optic disc, respectively), and microscopic fields measuring 200×250 μm^2^ were selected. Labeled ganglion cells were counted at 200× magnification in 4 central regions, 8 middle regions and 12 peripheral regions in the 4 quadrants of the retina. Corresponding regions from each retina of experimental and control groups were used for counting.

### Western blot analysis

Both the control and experimental retinas were homogenized in a radioimmunoprecipitation assay (RIPA) buffer consisting of 1% Triton X-100, 5% sodium dodecyl sulfate (SDS), 5% deoxycholic acid, 0.5 M Tris-HCl (pH 7.5), 10% glycerol, 1 mM ethylenediaminetetraacetic acid (EDTA), 1 mM phenylmethylsulfonyl fluoride (PMSF), 5 μg/ml aprotinin, 1 μg/ml leupeptin, 1 μg/ml pepstatin, 200 mM sodium orthovanadate and 200 mM sodium fluoride. The tissue extracts were incubated for 10 min on ice and clarified by centrifugation at 10,000 ***g*** for 25 min at 4°C. The total protein levels in the retinal extracts were measured using a standard bicinchoninic acid (BCA) assay (Pierce, Rockford, IL, USA). The retinal extracts (40 μg of total protein) were then resuspended in 5× sample buffer (60 mM Tris-HCl, pH 7.4, 25% glycerol, 2% SDS, 14.4 mM 2-mercaptoethanol, 0.1% Bromophenol Blue) at a 4:1 ratio, boiled for 5 min and resolved using an SDS-polyacrylamide gel electrophoresis (PAGE) procedure.

The proteins were then transferred onto a nitrocellulose membrane and the blots were stained with Ponseau S (Sigma-Aldrich, St Louis, MO, USA) to visualize the protein bands, and ensure equal protein loading and uniform transfer. The blots were washed and blocked for 45 min with 5% non-dried skim milk in Tris-buffered saline with Tween 20 (TBST; 20 mM Tris-HCl, pH 7.6, 137 mM NaCl and 0.1% Tween 20) and then probed for 24 h using antibodies against synaptophysin (Cell Signaling Technology), BDNF (Santa Cruz Biotechnology, Inc.), *p*-Akt, Akt (Cell Signaling Technology), *p*-CaMKII (Santa Cruz Biotechnology), *p*-CREB (Cell Signaling Technology), F-actin (Cell Signaling Technology), NMDAR1 (Cell Signaling Technology), NMDAR2B (Cell Signaling Technology) and glyceraldehyde-3-phosphate dehydrogenase (GAPDH; Sigma-Aldrich). The blots were then probed with horseradish peroxidase (HRP)-conjugated goat anti-rabbit secondary antibodies. Bound antibodies were detected using an enhanced chemiluminescence system (Amersham, Piscataway, NJ, USA) and X-ray film. The relative intensity was measured using an ImageMaster^®^ VDS (Pharmacia Biotech, Piscataway, NJ, USA); the fold changes in these protein levels are indicated below the blots. The results are representative of 5 independent experiments and all data are expressed as mean±s.d.

### Statistical analysis

All data are expressed as means±s.d. Comparisons between time points or with the controls were performed using the Student's *t*-test and multiple comparisons using the Scheffe's post hoc method. Differences with *P*<0.05 were considered statistically significant.

## References

[DMM037184C1] Agostinone J. and Di Polo A. (2015). Retinal ganglion cell dendrite pathology and synapse loss: Implications for glaucoma. *Prog. Brain Res.* 220, 199-216. 10.1016/bs.pbr.2015.04.012 26497792

[DMM037184C2] Binley K. E., Ng W. S., Barde Y. A., Song B. and Morgan J. E. (2016). Brain-derived neurotrophic factor prevents dendritic retraction of adult mouse retinal ganglion cells. *Eur. J. Neurosci.* 44, 2028-2039. 10.1111/ejn.13295 27285957 PMC4988502

[DMM037184C3] Bustos F. J., Jury N., Martinez P., Ampuero E., Campos M., Abarzua S., Jaramillo K., Ibing S., Mardones M. D., Haensgen H. et al. (2017). NMDA receptor subunit composition controls dendritogenesis of hippocampal neurons through CAMKII, CREB-P, and H3K27ac. *J. Cell. Physiol.* 232, 3677-3692. 10.1002/jcp.25843 28160495

[DMM037184C4] Carmignoto G., Maffei L., Candeo P., Canella R. and Comelli C. (1989). Effect of NGF on the survival of rat retinal ganglion cells following optic nerve section. *J. Neurosci.* 9, 1263-1272. 10.1523/JNEUROSCI.09-04-01263.1989 2467970 PMC6569868

[DMM037184C5] Chrysostomou V., Galic S., van Wijngaarden P., Trounce I. A., Steinberg G. R. and Crowston J. G. (2016). Exercise reverses age-related vulnerability of the retina to injury by preventing complement-mediated synapse elimination via a BDNF-dependent pathway. *Aging Cell* 15, 1082-1091. 10.1111/acel.12512 27613664 PMC5114604

[DMM037184C6] Clarke D. B., Bray G. M. and Aguayo A. J. (1998). Prolonged administration of NT-4/5 fails to rescue most axotomized retinal ganglion cells in adult rats. *Vision Res.* 38, 1517-1524. 10.1016/S0042-6989(97)00341-6 9667016

[DMM037184C7] Clarkson A. N., Parker K., Nilsson M., Walker F. R. and Gowing E. K. (2015). Combined ampakine and BDNF treatments enhance poststroke functional recovery in aged mice via AKT-CREB signaling. *J. Cereb. Blood Flow Metab.* 35, 1272-1279. 10.1038/jcbfm.2015.33 25757752 PMC4528000

[DMM037184C8] Cohen-Cory S. and Fraser S. E. (1995). Effects of brain-derived neurotrophic factor on optic axon branching and remodelling in vivo. *Nature* 378, 192-196. 10.1038/378192a0 7477323

[DMM037184C9] Dancause N., Barbay S., Frost S. B., Plautz E. J., Chen D., Zoubina E. V., Stowe A. M. and Nudo R. J. (2005). Extensive cortical rewiring after brain injury. *J. Neurosci.* 25, 10167-10179. 10.1523/JNEUROSCI.3256-05.2005 16267224 PMC6725801

[DMM037184C10] Evans G. J. (2007). Synaptic signalling in cerebellar plasticity. *Biol. Cell* 99, 363-378. 10.1042/BC20070010 17567263

[DMM037184C11] Garcia-Valenzuela E., Shareef S., Walsh J. and Sharma S. C. (1995). Programmed cell death of retinal ganglion cells during experimental glaucoma. *Exp. Eye Res.* 61, 33-44. 10.1016/S0014-4835(95)80056-5 7556468

[DMM037184C12] Jelsma T. N., Friedman H. H., Berkelaar M., Bray G. M. and Aguayo A. J. (1993). Different forms of the neurotrophin receptor trkB mRNA predominate in rat retina and optic nerve. *J. Neurobiol.* 24, 1207-1214. 10.1002/neu.480240907 8409978

[DMM037184C13] Jia J. M., Chen Q., Zhou Y., Miao S., Zheng J., Zhang C. and Xiong Z. Q. (2008). Brain-derived neurotrophic factor-tropomyosin-related kinase B signaling contributes to activity-dependent changes in synaptic proteins. *J. Biol. Chem.* 283, 21242-21250. 10.1074/jbc.M800282200 18474605 PMC3258936

[DMM037184C14] Johnson J. E., Barde Y. A., Schwab M. and Thoenen H. (1986). Brain-derived neurotrophic factor supports the survival of cultured rat retinal ganglion cells. *J. Neurosci.* 6, 3031-3038. 10.1523/JNEUROSCI.06-10-03031.1986 2876066 PMC6568792

[DMM037184C15] Kalesnykas G., Oglesby E. N., Zack D. J., Cone F. E., Steinhart M. R., Tian J., Pease M. E. and Quigley H. A. (2012). Retinal ganglion cell morphology after optic nerve crush and experimental glaucoma. *Invest. Ophthalmol. Vis. Sci.* 53, 3847-3857. 10.1167/iovs.12-9712 22589442 PMC3630905

[DMM037184C16] Kang H. and Schuman E. M. (1995). Long-lasting neurotrophin-induced enhancement of synaptic transmission in the adult hippocampus. *Science* 267, 1658-1662. 10.1126/science.7886457 7886457

[DMM037184C17] Kim H. S. and Park C. K. (2005). Retinal ganglion cell death is delayed by activation of retinal intrinsic cell survival program. *Brain Res.* 1057, 17-28. 10.1016/j.brainres.2005.07.005 16139821

[DMM037184C18] Kim H. S., Chang Y. I., Kim J. H. and Park C. K. (2007). Alteration of retinal intrinsic survival signal and effect of alpha2-adrenergic receptor agonist in the retina of the chronic ocular hypertension rat. *Vis. Neurosci.* 24, 127-139. 10.1017/S0952523807070150 17640403

[DMM037184C19] Ko M.-L., Hu D.-N., Ritch R., Sharma S. C. and Chen C. F. (2001). Patterns of retinal ganglion cell survival after brain-derived neurotrophic factor administration in hypertensive eyes of rats. *Neurosci. Lett.* 305, 139-142. 10.1016/S0304-3940(01)01830-4 11376903

[DMM037184C20] Kotaleski J. H. and Blackwell K. T. (2010). Modelling the molecular mechanisms of synaptic plasticity using systems biology approaches. *Nat. Rev. Neurosci.* 11, 239-251. 10.1038/nrn2807 20300102 PMC4831053

[DMM037184C21] Lau C. G. and Zukin R. S. (2007). NMDA receptor trafficking in synaptic plasticity and neuropsychiatric disorders. *Nat. Rev. Neurosci.* 8, 413-426. 10.1038/nrn2153 17514195

[DMM037184C22] Leal G., Comprido D. and Duarte C. B. (2014). BDNF-induced local protein synthesis and synaptic plasticity. *Neuropharmacology* 76, 639-656. 10.1016/j.neuropharm.2013.04.005 23602987

[DMM037184C23] Levkovitch-Verbin H., Quigley H. A., Martin K. R., Zack D. J., Pease M. E. and Valenta D. F. (2003). A model to study differences between primary and secondary degeneration of retinal ganglion cells in rats by partial optic nerve transection. *Invest. Ophthalmol. Vis. Sci.* 44, 3388-3393. 10.1167/iovs.02-0646 12882786

[DMM037184C24] Lu B. (2003). BDNF and activity-dependent synaptic modulation. *Learn. Mem.* 10, 86-98. 10.1101/lm.54603 12663747 PMC5479144

[DMM037184C25] Mansour-Robaey S., Clarke D. B., Wang Y. C., Bray G. M. and Aguayo A. J. (1994). Effects of ocular injury and administration of brain-derived neurotrophic factor on survival and regrowth of axotomized retinal ganglion cells. *Proc. Natl. Acad. Sci. USA* 91, 1632-1636. 10.1073/pnas.91.5.1632 8127857 PMC43217

[DMM037184C26] Maren S. and Baudry M. (1995). Properties and mechanisms of long-term synaptic plasticity in the mammalian brain: relationships to learning and memory. *Neurobiol. Learn. Mem.* 63, 1-18. 10.1006/nlme.1995.1001 7663875

[DMM037184C27] Mey J. and Thanos S. (1993). Intravitreal injections of neurotrophic factors support the survival of axotomized retinal ganglion cells in adult rats in vivo. *Brain Res.* 602, 304-317. 10.1016/0006-8993(93)90695-J 8448673

[DMM037184C28] Morgan J. E., Datta A. V., Erichsen J. T., Albon J. and Boulton M. E. (2006). Retinal ganglion cell remodelling in experimental glaucoma. *Adv. Exp. Med. Biol.* 572, 397-402. 10.1007/0-387-32442-9_56 17249602

[DMM037184C29] Papadopoulos C. M., Tsai S. Y., Alsbiei T., O'Brien T. E., Schwab M. E. and Kartje G. L. (2002). Functional recovery and neuroanatomical plasticity following middle cerebral artery occlusion and IN-1 antibody treatment in the adult rat. *Ann. Neurol.* 51, 433-441. 10.1002/ana.10144 11921049

[DMM037184C30] Park H.-Y., Kim J. H. and Park C. K. (2014). Alterations of the synapse of the inner retinal layers after chronic intraocular pressure elevation in glaucoma animal model. *Mol. Brain* 7, 53 10.1186/s13041-014-0053-2 25116810 PMC4237962

[DMM037184C31] Pascual M., Climent E. and Guerri C. (2001). BDNF induces glutamate release in cerebrocortical nerve terminals and in cortical astrocytes. *Neuroreport* 12, 2673-2677. 10.1097/00001756-200108280-00017 11522946

[DMM037184C32] Patterson S. L., Abel T., Deuel T. A., Martin K. C., Rose J. C. and Kandel E. R. (1996). Recombinant BDNF rescues deficits in basal synaptic transmission and hippocampal LTP in BDNF knockout mice. *Neuron* 16, 1137-1145. 10.1016/S0896-6273(00)80140-3 8663990

[DMM037184C33] Peinado-Ramon P., Salvador M., Villegas-Perez M. P. and Vidal-Sanz M. (1996). Effects of axotomy and intraocular administration of NT-4, NT-3, and brain-derived neurotrophic factor on the survival of adult rat retinal ganglion cells. A quantitative in vivo study. *Invest. Ophthalmol. Vis. Sci.* 37, 489-500. 8595949

[DMM037184C34] Perez M.-T. and Caminos E. (1995). Expression of brain-derived neurotrophic factor and of its functional receptor in neonatal and adult rat retina. *Neurosci. Lett.* 183, 96-99. 10.1016/0304-3940(94)11123-Z 7746496

[DMM037184C35] Quigley H. A. (1999). Neuronal death in glaucoma. *Prog. Retin. Eye Res.* 18, 39-57. 10.1016/S1350-9462(98)00014-7 9920498

[DMM037184C36] Quigley H. A., Addicks E. M., Green W. R. and Maumenee A. E. (1981). Optic nerve damage in human glaucoma. II. The site of injury and susceptibility to damage. *Arch. Ophthalmol.* 99, 635-649. 10.1001/archopht.1981.03930010635009 6164357

[DMM037184C37] Quigley H. A., Nickells R. W., Kerrigan L. A., Pease M. E., Thibault D. J. and Zack D. J. (1995). Retinal ganglion cell death in experimental glaucoma and after axotomy occurs by apoptosis. *Invest. Ophthalmol. Vis. Sci.* 36, 774-786. 7706025

[DMM037184C38] Riccio A. and Ginty D. D. (2002). What a privilege to reside at the synapse: NMDA receptor signaling to CREB. *Nat. Neurosci.* 5, 389-390. 10.1038/nn0502-389 11976696

[DMM037184C39] Schwab M. E. (1996). Structural plasticity of the adult CNS. Negative control by neurite growth inhibitory signals. *Int. J. Dev. Neurosci.* 14, 379-385. 10.1016/0736-5748(96)00024-X 8884371

[DMM037184C40] Shou T., Liu J., Wang W., Zhou Y. and Zhao K. (2003). Differential dendritic shrinkage of alpha and beta retinal ganglion cells in cats with chronic glaucoma. *Invest. Ophthalmol. Vis. Sci.* 44, 3005-3010. 10.1167/iovs.02-0620 12824245

[DMM037184C41] Thanos S. and Vanselow J. (1989). The effect of central and peripheral neuroglia on the regeneration of the optic nerve. *Fortschr Ophthalmol* 86, 172-175. 2786831

[DMM037184C42] Unoki K. and LaVail M. M. (1994). Protection of the rat retina from ischemic injury by brain-derived neurotrophic factor, ciliary neurotrophic factor, and basic fibroblast growth factor. *Invest. Ophthalmol. Vis. Sci.* 35, 907-915. 8125754

[DMM037184C43] Weber A. J. and Harman C. D. (2005). Structure-function relations of parasol cells in the normal and glaucomatous primate retina. *Invest. Ophthalmol. Vis. Sci.* 46, 3197-3207. 10.1167/iovs.04-0834 16123419 PMC1351226

[DMM037184C44] Weber A. J., Kaufman P. L. and Hubbard W. C. (1998). Morphology of single ganglion cells in the glaucomatous primate retina. *Invest. Ophthalmol. Vis. Sci.* 39, 2304-2320. 9804139

[DMM037184C45] Yan X., Liu J., Ye Z., Huang J., He F., Xiao W., Hu X. and Luo Z. (2016). CaMKII-mediated CREB phosphorylation is involved in Ca2+-Induced BDNF mRNA transcription and neurite outgrowth promoted by electrical stimulation. *PLoS ONE* 11, e0162784. 27611779 10.1371/journal.pone.0162784 PMC5017744

[DMM037184C46] Zhong P., Liu Y., Hu Y., Wang T., Zhao Y.-P. and Liu Q.-S. (2015). BDNF interacts with endocannabinoids to regulate cocaine-induced synaptic plasticity in mouse midbrain dopamine neurons. *J. Neurosci.* 35, 4469-4481. 10.1523/JNEUROSCI.2924-14.2015 25762688 PMC4355208

